# Uridine treatment prevents myocardial injury in rat models of acute ischemia and ischemia/reperfusion by activating the mitochondrial ATP-dependent potassium channel

**DOI:** 10.1038/s41598-021-96562-7

**Published:** 2021-08-20

**Authors:** Irina B. Krylova, Elena N. Selina, Valentina V. Bulion, Olga M. Rodionova, Natalia R. Evdokimova, Natalia V. Belosludtseva, Maria I. Shigaeva, Galina D. Mironova

**Affiliations:** 1 Department of Neuropharmacology, Federal State Budgetary Scientific Institution, Institute of Experimental Medicine, St. Petersburg, Russia 197376; 2 Laboratory of Mitochondrial Transport, Institute of Theoretical and Experimental Biophysics of Russian Academy of Sciences, Pushchino, Moscow Region, Russia 142290

**Keywords:** Biochemistry, Cardiology, Pathogenesis

## Abstract

The effect of uridine on the myocardial ischemic and reperfusion injury was investigated. A possible mechanism of its cardioprotective action was established. Two rat models were used: (1) acute myocardial ischemia induced by occlusion of the left coronary artery for 60 min; and (2) myocardial ischemia/reperfusion with 30-min ischemia and 120-min reperfusion. In both models, treatment with uridine (30 mg/kg) prevented a decrease in cell energy supply and in the activity of the antioxidant system, as well as an increase in the level of lipid hydroperoxides and diene conjugates. This led to a reduction of the necrosis zone in the myocardium and disturbances in the heart rhythm. The blocker of the mitochondrial ATP-dependent potassium (mitoK_ATP_) channel 5-hydroxydecanoate limited the positive effects of uridine. The data indicate that the cardioprotective action of uridine may be related to the activation of the mitoK_ATP_ channel. Intravenously injected uridine was more rapidly eliminated from the blood in hypoxia than in normoxia, and the level of the mitoK_ATP_ channel activator UDP in the myocardium after uridine administration increased. The results suggest that the use of uridine can be a potentially effective approach to the management of cardiovascular diseases.

## Introduction

Despite some progress in the prevention and treatment of myocardial coronary artery disease, this pathology remains one of the main causes of disability and mortality in the world^1^. Hypoxia occurring in acute myocardial infarction leads to the reduction of mitochondrial respiratory chain complexes due to the lack of molecular oxygen, which is accompanied by a rapid depletion of high-energy phosphates^2^ and the accumulation of reactive oxygen species (ROS) in cardiomyocytes^3^. As a result, the dysfunction of energy-dependent ion pumps, metabolic acidosis, and oxidative stress can develop in the heart tissue^4,5^. The latter occurs when antioxidants are unable to remove excessive ROS, which leads to the destruction of integrity of cellular membranes. Prolonged and severe ischemia can induce irreversible damage, which culminates in cell death and the loss of viable myocardium^2,4^.

The European Society of Cardiology guidelines emphasize the importance of early reperfusion therapy of myocardial infarction^6^. Thrombolytic therapy and primary percutaneous coronary intervention made it possible to reduce significantly the risk of acute myocardial ischemic injury and decrease mortality in the case of the acute coronary syndrome. However, these interventions on their own can trigger metabolic alterations, such as intracellular calcium overloading and oxidative stress, which result in pathological changes in the structure and functioning of cardiomyocytes, a decrease in the myocardial contractile function, and the development of reperfusion arrhythmias^2,4^. The reperfusion injury of myocardium in itself can be the cause of death and requires novel therapeutic interventions.

Due to the high relevance of the problem of preserving the myocardial function, considerable efforts are aimed at the development of new pharmacological approaches to improve functional recovery and to limit the extent of tissue injury caused by ischemia/reperfusion (I/R). In this context, a problem of particular importance is the development of an integrated approach to the treatment of acute myocardial infarction and its reperfusion complications. Since acute myocardial infarction is a multifactor disease that causes cardiomyocyte death via multiple intracellular mechanisms^7^, the multitarget therapeutic strategy may be most effective. Under this approach, it is of great interest to find the ways of pharmacological control of the activity of the mitochondrial ATP-dependent potassium (mitoK_ATP_) channel, which plays an important role in the protection of the myocardium from ischemic damage and can be the final effector in the cardioprotective effects of ischemic preconditioning^5,8,9^. Our previous studies have demonstrated that the intermittent hypoxic training of animals significantly increased the functional activity of this channel in isolated mitochondria^10^. Many synthetic activators of the mitoK_ATP_ channel were found to display cardioprotective effects in experimental research^5,9^. Their use in animal models promotes the preservation of the structural and functional integrity of mitochondria and heart tissue, the maintenance of ATP synthesis and, correspondingly, an increase in myocardial resistance under conditions of ischemia and reperfusion^11^. However, the most promising therapeutic approach would be the use of natural metabolic compounds, which are regulators of endogenous defense mechanisms and can be involved in the adaptation of the myocardium to pathological conditions. Among these activators are, for example, the sex hormones β-estradiol and testosterone^12,13^.

Earlier, we have found that uridine-5′-diphosphate (UDP) is an effective metabolic activator of the mitoK_ATP_ channel^14^. This phosphonucleotide at micromolar concentrations activates both the channel protein reconstructed into artificial membranes and the native channels localized in the inner mitochondrial membrane. However, the use of UDP for the channel activation is ineffective, since it is incapable of penetrating the cell membrane^15^ and, hence, affect the channels located in mitochondria. Therefore, a potential tool for the prevention and treatment of myocardial ischemic and I/R injury may be the metabolic precursor of UDP, pyrimidine nucleoside uridine, as it is transported into the cell where it serves as a source of UDP synthesis^16^.

The pharmacological activity of uridine and its nucleotides toward cardiovascular diseases is still poorly understood^17^. We have shown previously that uridine prevents the depression of the contractile function of the ischemic myocardium in isolated hearts^18^ and the development of myocardial stunning after postischemic reperfusion^19^. In addition, uridine exhibits antihypoxic activity^20,21^, reduces the posthypoxic pulmonary edema^22^, prevents oxidative stress during acute bacterial inflammation^23^, and increases the physical endurance of rats with low resistance to acute hypobaric hypoxia^24^.

The aim of the present work was to investigate the cardioprotective effect of uridine in vivo in the models of acute myocardial ischemia (AMI) and myocardial I/R in rats and to reveal whether the mitoK_ATP_ channel can be involved in the mechanism of action of the nucleoside.

## Results

### Elimination of exogenous uridine from the rat blood serum

**Figure 1:**
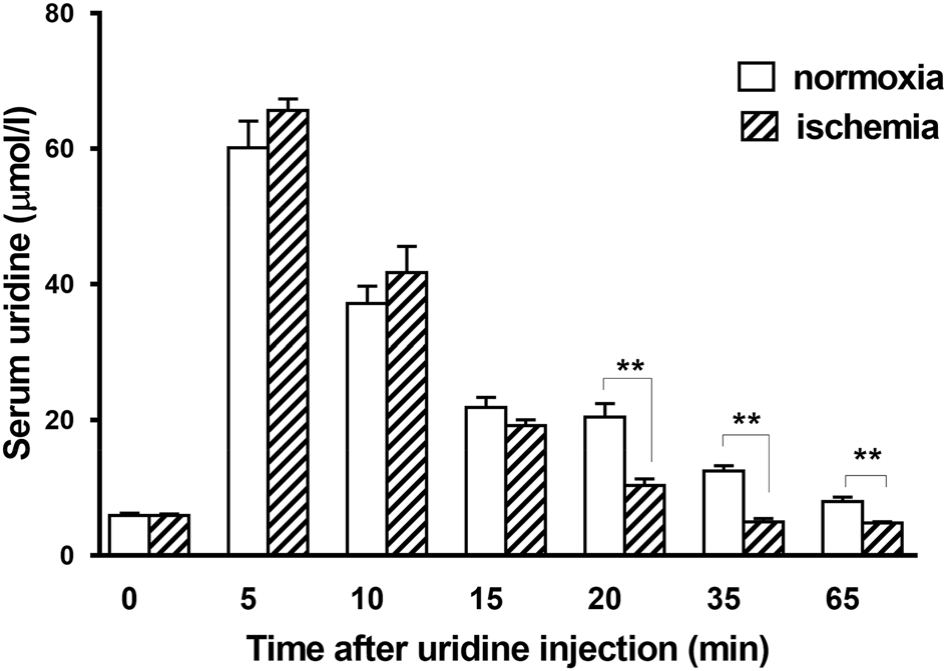
Levels of serum uridine after the intravenous administration of the nucleoside to rats in the control group (normoxia, *n* = 6) and in the group of animals with acute myocardial ischemia (AMI, *n* = 6). Uridine was given at a dose of 30 mg/kg five min before AMI. The determination of uridine in rat blood serum by HPLC was carried out at the following time points: 0 (background level), 5, 10, 15, 30, and 65 min after uridine administration. Data shown are the mean ± SD (*n* = 6). **p < 0.01 (vs. normoxia group).

Figure 1 shows that the background levels of uridine in control rats and rats with AMI were the same (5.92 ± 0.31 μmol/L and 5.93 ± 0.19 μmol/L, correspondingly). It should be noted that the results obtained are consistent with the literature data, which demonstrate that serum uridine is normally maintained at a relatively constant level in a range of 5.4–5.7 µmol/L^25,26^. Five minutes after the i.v. administration of the nucleoside to rats of both groups, its level in blood serum increased 11 times compared to the background level and amounted to 65.3 ± 3.6 μmol/L in the control and 63.9 ± 2.3 μmol/L in the AMI group (Fig. 1). In both groups, after a sharp increase in the concentration of serum uridine, its gradual elimination from the blood was observed. Within the first 15 min after the injection, the rate of uridine elimination was the same in two groups, and as a result, its level in the blood serum of animals in both groups did not differ. However, by 20 min of the experiment, the serum uridine level in rats with AMI sharply decreased to 10.3 ± 0.9 μmol/L; and by 35 min, it was even lower than the background level and remained approximately the same until the end of the experiment. In control rats, 20 min after the administration of uridine, its level was two times higher than in rats with the LCA occlusion. The elevated level of uridine in the control remained up to 65 min. Thus, intravenously administered uridine is more rapidly removed from the blood during AMI than under normal conditions and, hence, can be consumed by tissues at a higher rate.

### The levels of UDP and UTP in the rat myocardium after uridine administration

The content of UDP and UTP in the myocardium of normal rats was significantly lower than the level of ATP. As follows from Table 1, the concentration of UTP was three times higher than that of UDP. At 65 min after the intravenous administration of uridine to control rats, the concentration of UDP and UTP in the myocardium increased 2.0 and 2.2 times, respectively. At the same time, the quantitative UDP/UTP ratio in the tissue remained approximately the same.

**Table 1:** Concentration of UDP and UTP (nmol/g wet tissue) in the myocardium of control rats and rats with AMI 65 min after administration of uridine.

Groups	*n*	UDP	UTP	UDP + UTP
Control (intact)	8	37.8 ± 1.9	113.0 ± 7.9	150.8 ± 12.8
Control + uridine	6	74.4 ± 3.6***	248.6 ± 18.4***	323.0 ± 21.9***
AMI	5	52.0 ± 6.1*^,§^	67.8 ± 8.4**^,§§§^	119.8 ± 9.7*^,§§§^
AMI + uridine	5	76.6 ± 6.8***^,##^	301.2 ± 32.7***^,###^	377.8 ± 27.1***^,###^

AMI, acute myocardial ischemia; UDP, uridine-diphosphate; UTP, uridine-triphosphateAll data are the mean ± SD (*n* = 5–8).

*p < 0.05 versus the control group; **p < 0.01 versus the control group, ***p < 0.001 versus the control group; ^§^*p* < 0.05 versus the control + uridine group, ^§§§^*p* < 0.001 versus the control + uridine group; ^##^*p* < 0.01 versus AMI group; ^###^*p* < 0.001 versus AMI group. Significance of differences was determined by a one-way ANOVA followed by the Turkey’s multiple comparison Post Hoc test.

At 60 min after the onset of AMI, the UDP level in the myocardium increased by 38%, and the UTP content decreased by 34% compared with the corresponding concentrations in control animals. One can assume that the increase in the UDP concentration is the result of enhanced decay of UTP, which occurs during ischemia and can play an adaptive role. In addition, intracellular UTP was found to be released in the circulation during cardiac ischemia^27^.

Uridine administration 5 min before the onset of AMI led to an increase in the UDP level in the myocardium by a factor of 1.5 compared to that observed 60 min after the onset of AMI without the injection of the drug. At the same time, the content of UTP increased 4.4 times, which can be related to its resynthesis from uridine and the restriction of its decomposition during AMI due to the anti-ischemic action of uridine.

### The energy-saving effect of uridine

The occlusion of the LCA for 60 min led to the disorder of energy metabolism in the ischemic myocardium (Table 2). The ATP content decreased by 35% compared with that in sham-operated animals. A more pronounced decrease (by 59%) in the CrP concentration was observed. The treatment with uridine prevented a deficiency of macroergic compounds in the myocardium of rats with AMI, and the concentration of ATP and CrP became similar to that in sham-operated rats. The blocker of the mitoK_ATP_ channel 5-HD administered 5 min before uridine prevented the energy-saving effect of the nucleoside, and the levels of ATP and CrP were the same as in rats with AMI.

**Table 2:** Effect of uridine on the level of macroergic compounds, lipid peroxidation and antioxidant status in the rat myocardium in acute ischemia (60 min).

Groups	*n*	ATP (µmol/g wet tissue)	CrP (µmol/g wet tissue)	LPOs (OD_532_)	SOD activity (U/mg pr)	GSH (μmol/g wet tissue)
Sham-operated	8	2.54 ± 0.15	6.63 ± 0.18	0.070 ± 0.003	4.54 ± 0.04	34.37 ± 0.62
AMI	10	1.65 ± 0.15***	2.75 ± 0.13***	0.138 ± 0.014***	3.26 ± 0.02***	23.99 ± 1.02***
AMI + uridine	10	2.81 ± 0.13^###^	6.56 ± 0.26^###^	0.077 ± 0.003^###^	3.98 ± 0.10***^,###^	33.61 ± 1.73^###^
AMI + uridine + 5-HD	10	1.41 ± 0.05***^,†††^	2.89 ± 0.29***^,†††^	0.124 ± 0.005***^,†††^	3.30 ± 0.08***^,†††^	20.75 ± 1.25***^,†††^

AMI acute myocardial ischemia, 5-HD 5-hydroxydecanoate, CrP creatine phosphate, LPOs lipid hydroperoxides, SOD superoxide dismutase, GSH reduced glutathione. All data are mean ± SEM (*n* = 8–10).

***p < 0.001 versus sham-operated group; ^##^*p* < 0.01 versus AMI group, ^###^*p* < 0.001 versus AMI group; ^†††^*p* < 0.001 versus AMI + uridine group. Significance of differences was determined by a one-way ANOVA followed by the Turkey’s multiple comparison Post Hoc test*.*

Myocardial I/R also caused a sharp decrease in the level of macroergic compounds in the tissue (Table 3). The ATP level in the myocardium 120 min after the onset of reperfusion was 60% lower than that in sham-operated animals. Treatment with uridine preserved ATP in the myocardium, and its concentration after reperfusion was close to that in sham-operated rats. The positive effect of uridine was partially eliminated by 5-HD. The CrP level in control rats with I/R decreased by 67% by 120 min of reperfusion compared with that in sham-operated animals. Uridine treatment increased the amount of CrP twofold in comparison with the control (I/R) group. 5-HD decreased the effect of uridine by 39%.

**Table 3:** Effect of uridine on ATP and CrP concentrations, lipid peroxidation and the antioxidant status in the rat myocardium after 120 min of reperfusion.

Groups	*n*	ATP (µmol/g wet tissue)	CrP (µmol/g wet tissue)	LPOs (OD_532_)	DC (OD_232_)	SOD activity (U/mg pr)	GSH (μmol/g wet tissue)
Sham-operated	8	2.77 ± 0.26	6.22 ± 0.36	0.068 ± 0.002	6.50 ± 0.41	4.72 ± 0.11	33.48 ± 0.63
I/R	10	1.15 ± 0.24***	2.04 ± 0.62***	0.096 ± 0.004***	10.55 ± 0.61***	4.03 ± 0.12***	25.49 ± 0.78***
I/R + uridine	10	2.50 ± 0.14^###^	4.71 ± 0.29 ^###^	0.063 ± 0.005^###^	7.69 ± 0.52^##^	4.67 ± 0.09^###^	31.65 ± 0.97^###^
I/R + uridine + 5-HD	9	1.46 ± 0.12***^,††^	2.89 ± 0.29***^,†^	0.084 ± 0.004^††^	11.12 ± 0.68***^, †††^	4.10 ± 0.20**^, ††^	28.28 ± 0.78***^,†^

I/R ischemia/reperfusion, 5-HD 5-hydroxydecanoate, CrP creatine phosphate, LPOs lipid hydroperoxides, DC diene conjugates, SOD superoxide dismutase, GSH reduced glutathione. All data are mean ± SEM (*n* = 8–10).

**p < 0.01 versus sham-operated group; ***p < 0.001 versus sham-operated group; ^##^*p* < 0.01 versus I/R group, ^###^*p* < 0.001 versus I/R group; ^†^*p* < 0.05 versus I/R + uridine group; ^††^*p* < 0.01 versus I/R + uridine group; ^†††^*p* < 0.001 versus I/R + uridine group. Significance of differences was determined by a one-way ANOVA followed by the Turkey’s multiple comparison Post Hoc test.

### Effect of uridine on lipid peroxidation and the antioxidant system

A deficiency of macroergic compounds in the ischemic myocardium was accompanied by enhanced lipid peroxidation. At 60 min after the occlusion, the level of lipid peroxides (LPOs) increased by 97% (Table 2). At the same time, the antioxidant system (AOS) was suppressed: the activity of superoxide dismutase (SOD) decreased by 28% and the amount of reduced glutathione (GSH) decreased by 30%.

The administration of uridine to animals 5 min before the ligation of the LCA completely prevented an increase in the production of LPOs and a decline in the level of GSH. The drug also limited a decrease in SOD activity. The data obtained indicate that uridine reduces the disorders in oxidative metabolism in the ischemic myocardium and restores the balance between the process of lipid peroxidation and the activity of AOS, which is important for maintaining the intracellular redox homeostasis during ischemia. 5-HD administered 5 min before uridine eliminated the protective effect of the nucleoside.

Postischemic reperfusion, similarly to acute ischemia, markedly increased lipid peroxidation and suppressed the antioxidant activity in the myocardium. At 120 min of reperfusion, the concentrations of the markers of oxidative stress LPOs and diene conjugates (DC) increased by 41% and 62%, respectively (Table 3). In parallel, a significant decrease in the activity of SOD and GSH was observed. The treatment with uridine completely prevented a reperfusion-induced increase in the amount of LPOs and reduced the formation of DC by 27% compared with the control. The nucleoside abolished the decline in SOD activity and increased the GSH level in the myocardium of rats with I/R to the level in sham-operated animals. The protective effect of uridine towards lipid peroxidation and antioxidant activity was diminished by simultaneous treatment with the inhibitor of the mitoK_ATP_ channel 5-HD.

### Anti-ischemic action of uridine

#### Effect of uridine on the size of the ischemic zone in AMI

**Figure 2:**
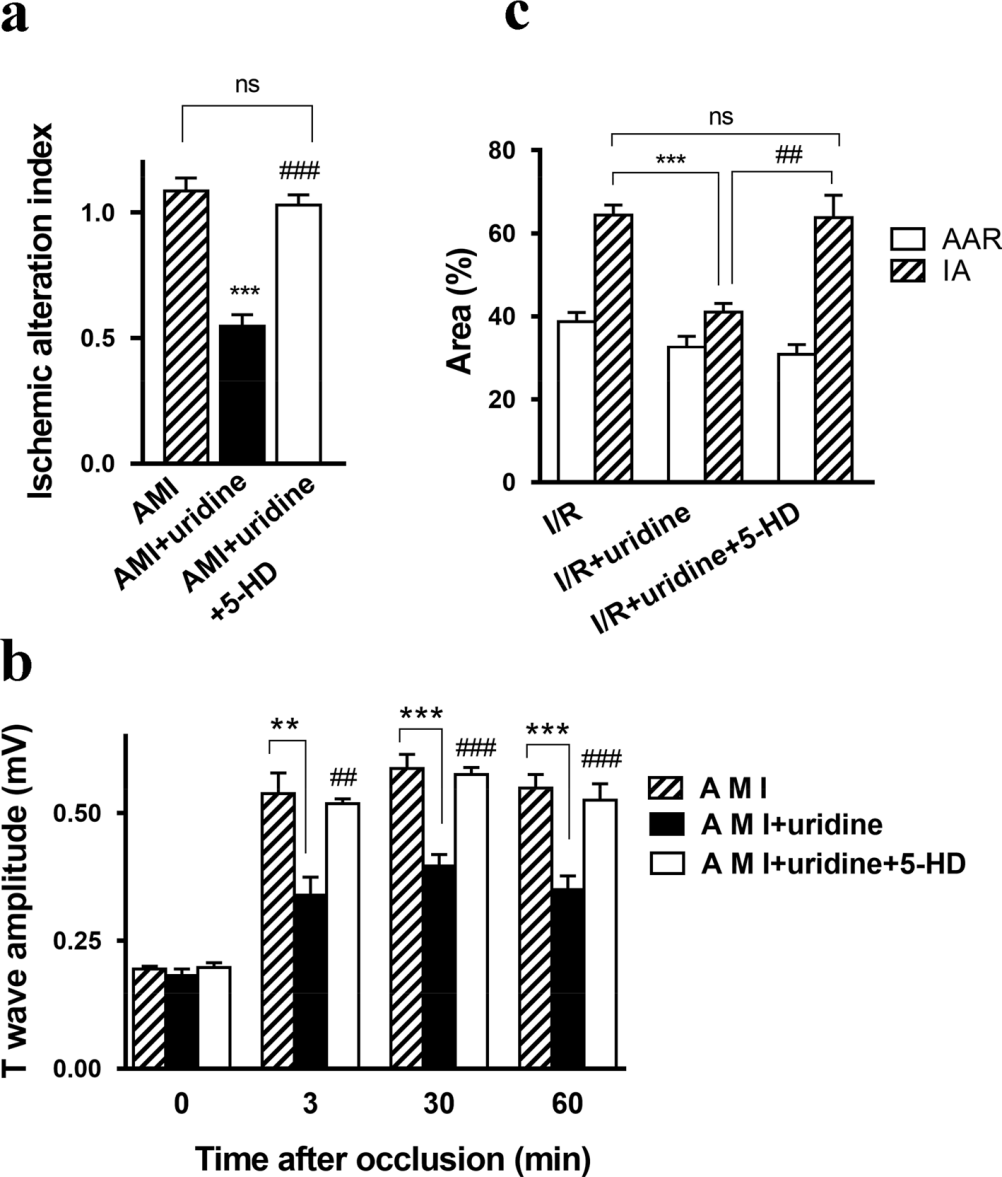
Anti-ischemic effect of uridine treatment in the AMI and I/R models. (**a**) Size of the ischemic alteration zone and (**b**) T-wave amplitude on the EEG in the AMI model. AMI (*n* = 15), AMI + uridine (*n* = 6), AMI + uridine + 5-HD (*n* = 6). (**c**) Infarct area in the rat myocardium after 120 min of reperfusion in the I/R model. Abbreviations: AMI, acute myocardial ischemia; I/R, ischemia/reperfusion; 5-HD, 5-hydroxydecanoate; IA, infarct area; AAR, area at risk; LCA, the left coronary artery. The AAR was expressed as a percentage of the total area of the left ventricle, and the IA, as a percentage of the AAR. I/R (*n* = 9), I/R + uridine (*n* = 5), I/R + uridine + 5-HD (*n* = 6). All data are the mean ± SD. **p < 0.01 versus AMI (or I/R) group, ***p < 0.001 versus AMI (or I/R) group, ^##^*p* < 0.01 versus AMI (or I/R) + uridine group, ^###^*p* < 0.001 versus AMI (or I/R) + uridine group.

By 60 min after the LCA occlusion, the activity of glycogen phosphorylase in cardiomyocytes markedly decreased, indicating damage to the left myocardial ventricle and the formation of the ischemic alteration zone. The size of the zone at this time point was 30–60% of the total volume of the left ventricle. The ischemic alteration index (IAI) in control animals with AMI was 1.085 ± 0.071 (Fig. 2a). The treatment with uridine 5 min before the onset of ischemia induced a reduction in the IAI to 0.588 ± 0.056. A preliminary blockade of the mitoK_ATP_ channel with 5-HD almost completely eliminated the positive effect of the nucleoside. According to the literature data^28^, 5-HD alone does not affect the size of the myocardial alteration zone.

#### Effect of uridine on the amplitude of the T-wave

AMI induced a pronounced increase in the amplitude of the T-wave on the ECG (Fig. 2b, Supplementary Fig. S1 online), which reflects the extent of ischemic myocardial injury. After 3-min occlusion of the LCA, the amplitude increased 2.5 times compared to the initial value and remained at this level until the end of the observation period (60 min). Preliminary administration of uridine decreased the ischemia-induced elevation of the T-wave amplitude. Its positive effect was completely blocked by 5-HD.

#### Effect of uridine on the formation of the infarct area during myocardial I/R

I/R led to the formation of an area at risk (AAR) in all experimental animals, which was approximately 32–39% of the volume of the left ventricle. In control animals with I/R, the infarct area (IA) was 64.4 ± 2.5% of the AAR (Fig. 2c). The double administration of uridine 30 min before ischemia and 5 min before reperfusion decreased the IA by 36%. 5-HD given 5 min before each injection of uridine completely prevented the manifestation of its positive effect.

### Anti-arrhythmic effect of uridine

#### Influence of uridine on early occlusion-induced arrhythmias

**Figure 3:**
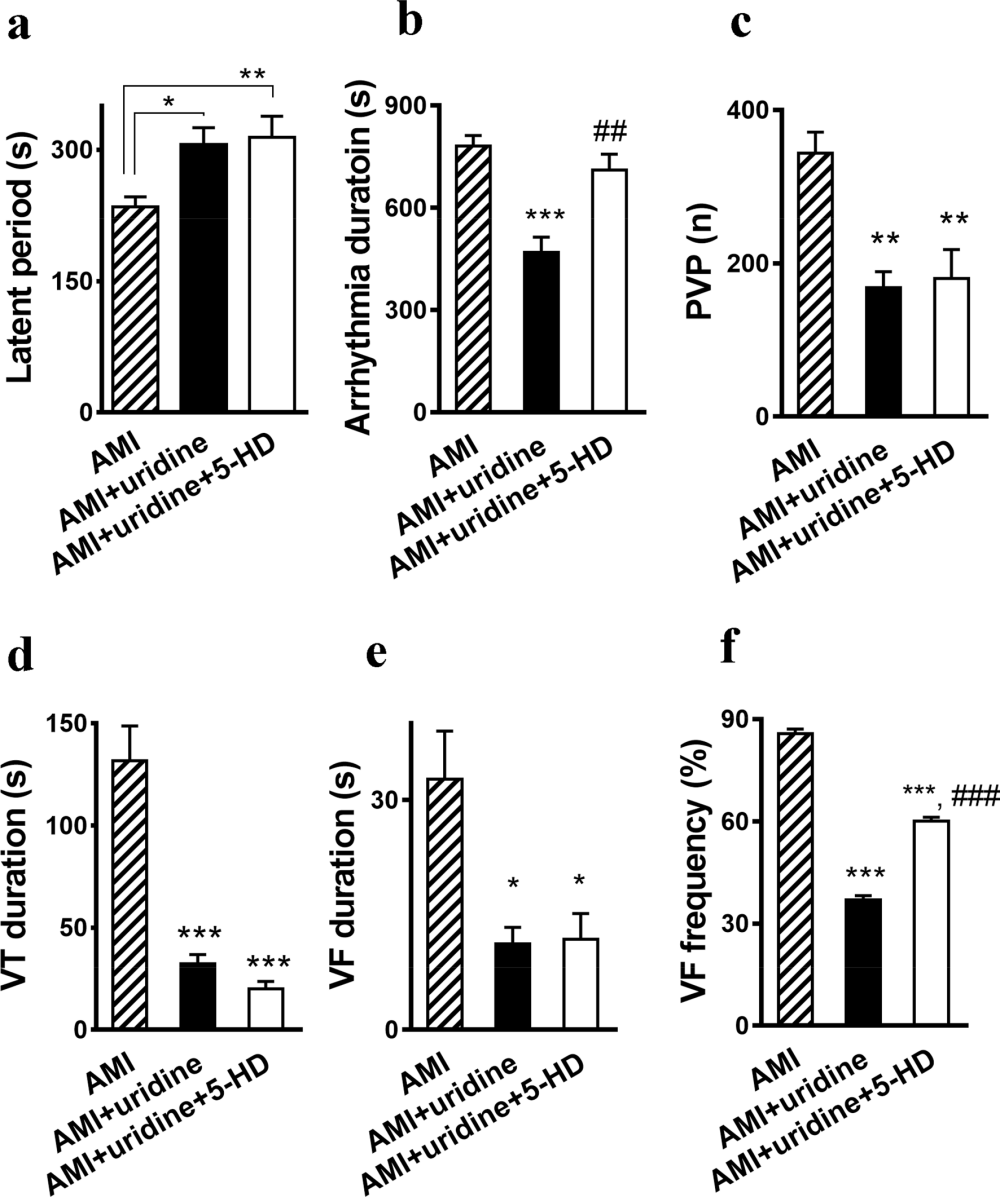
Effect of uridine on early postocclusion arrhythmia. (**a**) A latent period between the LCA occlusion and the first episode of arrhythmia and (**b**) the duration of arrhythmia. AMI (*n* = 11), AMI + uridine (*n* = 7), AMI + uridine + 5-HD (*n* = 7). Effect of uridine on the arrhythmia manifestation: number of PVP (**c**), duration of VT (**d**), duration of VF (**e**), frequency of VF (**f**). Abbreviations: AMI, acute myocardial ischemia; 5-HD, 5-hydroxydecanoate; PVP, premature ventricular beats; VT, ventricular tachycardia; VF, ventricular fibrillation. All data (except F) are the mean ± SEM. *p < 0.05 versus AMI group, **p < 0.01 versus AMI group, ***p < 0.001 versus AMI group, ^##^*p* < 0.01 versus AMI + uridine group, ^###^*p* < 0.001 versus AMI + uridine group.

The LCA occlusion led to the development of early post-occlusion arrhythmias in all experimental groups. In control (AMI), the disturbances of the heart rhythm were observed in 100% of cases. The first episode of arrhythmia occurred 4–5 min after the LCA ligation (Fig. 3a). The average duration of arrhythmias was 14–16 min after which the normal sinus rhythm was restored. The arrhythmias were characterized by the occurrence of premature ventricular beats (PVB), ventricular tachycardia (VT), and ventricular fibrillation (VF) (Supplementary Fig. S2 online).

Uridine reduced the total duration of arrhythmia 1.8 times (Fig. 3в) and increased the latent period until the onset of the first episode of arrhythmia (Fig. 3a). It decreased the number of PVB from 344 ± 27 in the control to 180 ± 37 (Fig. 3c) and the duration of VT from 132 ± 17 s to 32 ± 5 s (Fig. 3d). The blockade of the mitoK_ATP_ channel by 5-HD did not significantly affect the manifestation of antiarrhythmic activity of uridine toward PVB and VT.

Along with the above positive effects of uridine, a significant antifibrillatory action of the drug was found. It decreased the duration of VF 2.75 times (Fig. 3e). Since the administration of 5-HD did not abolish this effect, the antifibrillatory activity of uridine is most likely not related to the activation of the mitoK_ATP_ channel. The nucleoside also reduced the incidence of VF up to 37% compared to 86% in the control (Fig. 3f). In this case, 5-HD partially prevented the effect of uridine, and the frequency of occurrence of VF increased to 60%.

#### Effect of uridine on reperfusion-induced arrhythmias

**Figure 4:**
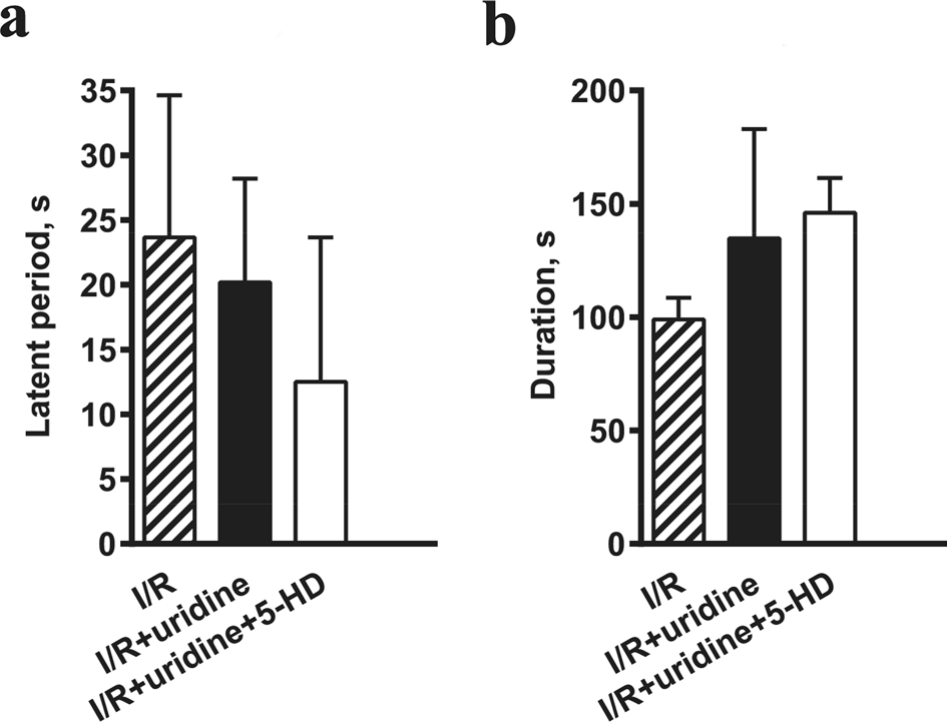
Effect of uridine on the onset of reperfusion-induced arrhythmias (**a**) and its duration (**b**). No statistical significance between I/R, I/R + uridine, and I/R + uridine + 5-HD groups was found.

The restoration of the coronary blood flow after 7-min myocardial ischemia resulted in the development of reperfusion disorders of heart rhythms in 70% of cases in the control group, in 67% of cases in the group of animals treated with uridine, and in 90% of cases in animals treated with a combination of uridine + 5-HD. Representative ECG recordings at the different time points in the I/R rat model are shown in Supplementary Fig. S3 online. The data on the onset and duration of reperfusion-induced arrhythmias are presented in Fig. 4a,b. As can be seen, uridine did not affect these parameters.

The main manifestations of reperfusion tachyarrhythmia were VT and VF (Supplementary Fig. S4 online). Table 4 presents the data on the influence of uridine on the frequency of occurrence, duration, and the number of episodes of VT. One can see that uridine had no effect on the incidence of VT. At the same time, it reduced the total duration and the number of episodes of VT three and two times, correspondingly. The blockade of the mitoK_ATP_ channel significantly decreased the protective effect of uridine toward VT.

**Table 4:** Effect of uridine on the ventricular tachycardia and ventricular fibrillation associated with myocardial I/R damage.

Groups	*n*	Ventricular tachycardia			Ventricular fibrillation (VF)	
		Frequency of occurrence (%)	Duration, s	Number of episodes	Frequency of occurrence (%)	Including lethal VF (%)
I/R	18	100	155 ± 27	155 ± 27	94% (17/18)	59% (10/17)
I/R + uridine	8	100	50 ± 12**	50 ± 12**	50% (4/8)*	75% (3/4)
I/R + uridine + 5-HD	10	100	118 ± 34^##^	118 ± 34^##^	90% (9/10)^#^	56% (5/9)

I/R ischemia/reperfusion, 5-HD 5-hydroxydecanoate. All data are the mean ± SD (*n* = 8–18).

*p < 0.05 versus I/R group, **p < 0.01 versus I/R group; ^#^*p* < 0.05 versus I/R + uridine group, ^##^*p* < 0.01 versus I/R + uridine group. Significance of differences was determined by a one-way ANOVA followed by the Turkey’s multiple comparison Post Hoc test.

The data on the influence of uridine on the reperfusion-induced VF are presented in Table 4. It reduced the frequency of occurrence of VF by 50% but increased the number of lethal VF among the animals with this type of arrhythmia. 5-HD given prior to uridine reduced the number of lethal VF to the values in untreated animals with I/R.

An integral assessment of the severity of reperfusion-induced disturbances of heart rhythm was carried out according to a 6-score system^29^. The results showed that the intensity of the reperfusion tachyarrhythmia corresponds to 5.2 ± 0.2 and 3.9 ± 0.6 scores in untreated animals and animals treated with uridine, respectively (Table 4). The data obtained suggest that uridine exhibits a moderate antiarrhythmic effect toward reperfusion-induced arrhythmias.

## Discussion

Using two rat models of myocardial injury, acute ischemia and ischemia–reperfusion, we have shown for the first time that treatment with uridine has a cardioprotective effect, which may be related to the activation of the mitoK_ATP_ channel by UDP. It should be noted that the use of the mitoK_ATP_ activator UDP as a pharmacological agent is complicated due to its instability and inability to penetrate through the cell membrane. At the same time, uridine is transported into the cell^16^ and could be a potential agent that increases the concentration of UDP in heart tissue.

In our experiments, acute ischemic injury of the myocardium resulted in the accelerated disappearance of uridine from the blood (Fig. 1). This is in agreement with the literature data that the perfusion of isolated rat hearts with a solution containing [H^3^] uridine shows the increased intensity of uridine uptake and its inclusion in uracil nucleotides in ischemia compared with normoxia^30^. It should be noted that, in our experiments, the period of the accelerated removal of uridine from the blood (15–30 min after the LCA occlusion) coincides with the most pronounced disturbances in energy metabolism in the myocardium^31^.

Simultaneously with the disappearance of uridine from the blood, the concentration of uridine nucleotides in the myocardial tissue changed. We found that the levels of UDP and UTP by 65 min after the administration of uridine to non-operated animals increased twofold (Table 1). In rats with AMI, the level of UDP in the myocardium increased, and the concentration of UTP decreased in comparison with the control (intact) group. Similar trends were noted in a culture of rat corticoencephalic cells under ischemic conditions^32^. It may be assumed that the increase in the level of UDP results from the disintegration of UTP to UDP provoked by ischemia. We do not exclude that this may be an endogenous protective mechanism leading to the activation of mitoK_ATP_. The low level of UTP in the ischemic myocardium can be also associated with its release from cardiomyocytes due to an increase in cell membrane permeability^27^. In rats treated with uridine, the increase in the UDP and UTP levels in the ischemic myocardium was more pronounced than that in rats without treatment (Table 1), indicating that exogenous uridine could be a source for the synthesis of UDP and UTP in cardiomyocytes under the experimental pathology.

As known, the main pathogenetic link of myocardial ischemic damage is a deficiency of macroergic compounds^33^. The results of our study showed that AMI and I/R markedly diminish the level of CrP and ATP in the rat myocardium (Tables 2 and 3). The treatment with uridine provided protection against the destabilization of energy metabolism by maintaining the level of ATP and CrP in the ischemic myocardium close to the norm. The combined treatment with uridine and the blocker of the mitoK_ATP_ channel 5-HD (5 mg/kg) prevented the energy-stabilizing effect of uridine, suggesting the involvement of the channel in the mechanism of cardioprotection. It should be noted that the used dose of 5-HD alone has no cardiotropic and side effects^28,34,35^. It may be assumed that the activation of the channel leads to the preservation of the structure and function of mitochondria and their ability to synthesize ATP. At the same time, one cannot exclude that the energy-saving effect of uridine can be related to its ability to activate glycogen synthesis and thereby the operation of the Krebs cycle through an increase in the synthesis of UTP and UDP-glucose^16,17,23^. This is also consistent with our earlier data indicating that exogenous uridine nucleotides increased the glycogen content and prevented the decomposition of pyruvate and at the same time did not increase ischemia-induced acidosis in the rat myocardium^31^.

It is well known that long-term hypoxia and I/R can lead to an excessive increase in lipid peroxidation and the depletion of the AOS, that is, the development of oxidative stress, which is an important factor in morphological and functional disorders in cardiomyocytes^6,36^. According to our data, the level of LPOs in the myocardium of rats with AMI and I/R increased by 97% and 41%, respectively. I/R led also to an increase in the content of DC by 62% compared with the sham-operated group. At the same time, the primary line of antioxidant defense, the activity of SOD, and the second line of defense, the level of GSH, were suppressed. Our results showed that uridine significantly limited the increase in lipid peroxidation and inhibition of AOS. The blocker of the mitoK_ATP_ channel 5-HD administered to rats 5 min before uridine eliminated (in the case of SOD) or significantly decreased (in the case of LPO, DC, GSH) the positive effect of uridine. Based on the results obtained on both models, we suggest that the antioxidant action of uridine, as well as its energy-stabilizing effect, are related to the activation of the mitoK_ATP_ channel.

As can be seen from the data obtained, the protection of heart tissue of rats with AMI and I/R from oxidative stress by uridine significantly reduces the size of the ischemic region in the myocardium (Fig. 2a,b). Furthermore, the anti-ischemic activity of the nucleoside also manifested itself in the reduction of the occlusion-induced increase in the amplitude of the T-wave, which is an electrophysiological indicator of the development of acute myocardial ischemia^37^. These positive effects of the drug were completely blocked by 5-HD.

It is well known that acute myocardial ischemia and postischemic restoration of the coronary blood flow are accompanied by the impairment of the electrical stability of the heart^38^. In our models, early ischemic arrhythmias developed in 100% of cases, and reperfusion arrhythmias in 70% of cases. They were characterized by the occurrence of PVB, VT, and VF. These heart rhythm disturbances develop due to a decrease in the duration of the membrane action potential and its regional heterogeneity. Extracellular K^+^ accumulation, which occurs during ischemia and ischemia/reperfusion, plays a key role in the initiation of these processes. Based on most parameters of assessing the effect of uridine on cardiac arrhythmias, we can conclude that the nucleoside has pronounced antiarrhythmic and antifibrillatory activity toward early ischemic and reperfusion arrhythmias. However, inhibitory analysis showed that uridine antiarrhythmic effect was not always associated with the activation of the mitoK_ATP_ channel. It is known that UDP is a non-selective agonist and can also affect sarcK_ATP_ channel activation, which can lead to an increase in the extracellular concentration of K^+^. It should be noted that the activation of the sarcK_ATP_ channel requires a higher concentration of UDP than that of the mitoK_ATP_ channel^14,39^. In hypoxic conditions, the positive effect of uridine can be also associated with the production of glycolytically derived ATP, which provides the functioning of ion channels and transporters in the cell membrane, including Na/K ATP-ase, the main regulator of the K/Na flow in the cell^40^. Therefore, the final effect of uridine on the heart rate during acute ischemia and I/R is likely to be the result of a complex integration of various processes that affect the electrical properties of cardiomyocytes. It can be explained partly by the opening of the mitoK_ATP_ channel and partly by the influence on the energy supply of the cell and the work of the ion channels of cell membranes.

A research group led by Prof. K. Garlid discovered that H_2_O_2_ is a metabolic opener of the mitoK_ATP_ channel^41^. It was found that the activation of the channel occurs in ischemic preconditioning, which is associated with a temporary increase in the degree of reduction of complexes of the electron transport chain and production of hydrogen peroxide in mitochondria^42^. The findings are also consistent with the data on the reversion of the cardioprotective effect of preconditioning by antioxidants^43,44^ and the mitoK_ATP_ blockers^28^. In addition, it was shown that intermittent hypoxic training of animals with low resistance to acute hypoxia leads both to an increase in the tolerance of rats to hypoxic exposure and the activation of mitoK_ATP_ channel^10^. Thus, the signaling role of ROS in cardioprotection may be mediated by an increase in the activity or expression level of mitoK_ATP_ channel^45,46^.

In contrast, prolonged and severe hypoxia is known to induce excessive lipid peroxidation in tissues, which leads to a depletion of the capacity of antioxidant systems and the development of oxidative stress^36^. Some studies indicate that an increase in the level of expression or activity of mitoK_ATP_ channel can protect tissues from oxidative damage by reducing excessive ROS production in mitochondria^8,35^.

It was found that succinate-driven reverse electron transport (RET) is one of the main sources of mitochondrial ROS upon ischemia/reperfusion injury^47^. It was shown that a driving force for RET is the mitochondrial membrane potential. Growing evidence is revealing that a slight decrease in the mitochondrial membrane potential may be applied as a therapeutic approach to diminish RET-driven ROS generation^47,48^. Notably, RET-induced ROS production was found to be attenuated by activation of mitochondrial potassium channels^49^.

In studies in vitro, the administration of pharmacological openers of mitoK_ATP_ channel was shown to induce a slight depolarization of the inner mitochondrial membrane^50,51^. It was also observed that mild mitochondrial uncoupling can be induced by the influx of potassium ions into mitochondria and the subsequent activation of the potassium recycling across the mitochondrial membrane^52^. In parallel, recent data suggest that the hypoxia-induced overexpression of mitochondrial uncoupling proteins also leads to a decrease in ROS production and protection of tissues from ischemic injury^53^.

At the same time, the activation of potassium recycling in mitochondria can prevent the hypoxia-induced calcium overload of the organelles, which leads to the opening of the mitochondrial permeability transition (MPT) pore and cell death^54,55^. The use of pharmacological openers of the mitochondrial potassium channels was reported to activate the potassium cycle in mitochondria and may prevent tissue damage under conditions of calcium overload^50,54–56^.

Thus, the data obtained in this work demonstrate that treatment with uridine attenuates myocardial injury and oxidative stress in acute ischemia and ischemia/reperfusion, which may be mediated by the activation of the mitoK_ATP_ channel. The findings are consistent with the data on the therapeutic effect of uridine in several pathologies associated with the development of hypoxia and oxidative stress, including cardiovascular diseases.

## Methods

### Animals

Male Wistar rats weighting 300–350 g were housed for two weeks before surgery under standard conditions at the room temperature of 18–22 °C, relative humidity 60–70%, and regular 12-h light–dark cycles. The animals received commercial pellets and water ad libitum. All surgery procedures were carried out on animals anesthetized with sodium pentobarbital (50 mg/kg) via intraperitoneal injection.

### Compliance with ethical standards

All manipulations with animals were performed in accordance with the European Convention for the Protection of Vertebrates used for experimental and other purposes (Strasbourg, 1986) and the principles of the Helsinki Declaration (2000). All the protocols were approved by the Ethics Committees at the Institute of Experimental Medicine and the Institute of Theoretical and Experimental Biophysics, Russian Academy of Sciences (Protocol No. 09 of 05.03.2019). The study was carried out in compliance with the ARRIVE guidelines.

### Acute myocardial ischemia (AMI) model

#### Surgical procedure

AMI was modeled by the occlusion of the descending branch of the left coronary artery (LCA) using a standard method^57^ with mechanical ventilation of the lungs through tracheostomy (Harvard rodent ventilator (USA), with a rate of 60 breaths/min, tidal volume of 2 ml/100 g). A significant elevation of the T-wave on electrocardiogram (ECG, lead II) indicated a successful modeling of AMI. ECG was recorded by a Schiller VET AT-1 device (Switzerland). The left femoral vein was catheterized for drug injections. Sham-operated rats underwent the same surgical procedures except that the suture around the LCA was not ligated.

#### Experimental protocol

Animals were randomly divided into 4 groups: 1—sham-operated rats (*n* = 9); 2—AMI + saline 5 min before occlusion (control, *n* = 17); 3—rats treated with uridine 30 mg/kg, intravenously (i.v.), 5 min prior to AMI (*n* = 18); 4—rats treated with uridine according to the same scheme + the inhibitor of the mitoK_ATP_ channel 5-hydroxydecanoate (5-HD) 5 mg/kg i.v., given 5 min prior to uridine (*n* = 18). Uridine and 5-HD were purchased from Sigma-Aldrich, St. Louis, MO, and dissolved in sterile saline. Sixty minutes after occlusion, the size of the ischemic alteration zone was determined, and biochemical assays of ischemic tissue were performed. Lead II ECG monitoring within the first 30 min after the LCA occlusion was fulfilled for the estimation of the heart rate. ECG measurements of T-wave elevation were taken before and 3, 30, and 60 min after the LCA occlusion.

### An ischemia–reperfusion (I/R) model

#### Surgical procedure

After left thoracotomy and pericardiotomy, a ligature (6–0 polypropylene suture) was placed around the descending branch of the LCA, and the ends of the suture were passed through a polyethylene tube to form a snare. Tight pulling of the suture and pressing to the epicardial surface resulted in the LCA occlusion and regional ischemia of the left ventricular. The suture was removed after 30 min of ischemia to allow reperfusion for 120 min. The effect of uridine on the reperfusion-induced arrhythmias was studied in a separate experiment in which the periods of acute ischemia and reperfusion were 7 and 30 min, correspondingly. ECG monitoring was performed from the first second of ischemia until the end of heart rhythm disorders or until the full cardiac arrest for the verification of myocardial ischemia and evaluation of the ventricular arrhythmias during myocardial I/R.

#### Experimental protocol

Animals were randomly divided into four groups: 1—sham-operated rats (*n* = 9); 2—I/R with saline 30 min before ischemia and 5 min before reperfusion (control, *n* = 19); 3—rats treated twice with i.v. injections of uridine at a dose of 30 mg/kg 30 min before ischemia and 5 min prior to reperfusion (*n* = 16); 4—rats treated with uridine 30 min before ischemia and 5 min prior to reperfusion + 5-HD at the dose of 5 mg/kg i.v. 5 min prior to each injection of uridine (*n* = 16). After the end of reperfusion, the area at risk and the zone of necrosis in the heart or biochemical changes in the myocardium were estimated.

To study a possible effect of uridine on the reperfusion arrhythmia, rats were randomly divided into three experimental groups: 1—rats injected with saline (1 ml/kg i.v.) 5 min before the LCA occlusion (control, *n* = 6); 2—rats treated with uridine (30 mg/kg i.v.) 5 min before the occlusion (*n* = 8); 3—rats injected with uridine (30 mg/kg i.v.) 5 min before the occlusion + 5-HD (5 mg/kg i.v.) 5 min prior the administration of uridine (*n* = 5).

### Measurement of the size of the infarct area

In the rat model of AMI, the area of early ischemic changes was visualized using the method of histochemical detection of glycogen phosphorylase activity^58^ 60 min after the LCA occlusion. The hearts were frozen at − 20 °C for further processing. Then, the heart was sliced transversely from the apex to the basal part of the left ventricle into four slices with a thickness of 2 mm. The ischemia zone was not stained. The size of this zone was determined by the planimetric method and expressed as the ischemic alteration index (IAI). The IAI was calculated by the following formula: IAI = ∑S_1-4_/linear magnification^2^ × heart weight, where S_1-4_ is the sum of the areas of all zones of ischemic damage in one heart^58^.

The infarct area (IA) and the area at risk (AAR) in the I/R model were determined after 120 min of reperfusion using the method of double staining^28^. A Canon A650IS camera and an MBS microscope (LOMO, Russia) were used to detect the AAR. The images were digitized using the Adobe Photoshop CC 2017 software (Adobe Systems Incorporated, San Jose, CA, USA). The AAR was expressed as a percentage of the total area of the histological section, and the IA, as a percentage of AAR.

### Oxidative stress-related parameters

The anterior wall of the left ventricle was quickly taken and frozen in liquid nitrogen. Then, a 10% homogenate in ice-cold phosphate buffer (50 mM, pH 7.4) was prepared and used for spectrophotometric assays of the levels of reduced glutathione (GSH)^59^, lipid hydroperoxides (LPO), diene conjugates (DC)^60^, and superoxide dismutase (SOD) activity^61^ with the use of a Unico2802S spectrophotometer (United products & Instruments, USA).

### Determination of UDP, UTP, ATP, and CrP in the myocardium by HPLC

The levels of UDP and UTP were determined in neutralized protein-free tissue extracts by HPLC using a Knauer chromatography system (Germany) with a ProPac PA 1 column (4 × 250 mm, 10 μm) (Sigma-Aldrich, St. Louis, MO) and ProPac PA 1 pre-column (4 × 50 mm, 10 μm) (Sigma-Aldrich, St. Louis, MO). The detection was carried out at a wavelength of 254 nm. The content of nucleotides in samples was assessed by the external standard method using the 1.52.0.0 software program for Windows.

ATP and CrP were separated by ion-pair reverse-phase HPLC according to published protocols^62^ with minor modifications using Beckman System Gold (USA) with a Saphire 600 UV detector (Ecom, Czechia) and an RP 18 column. ATP was detected at 254 nm, CrP at 210 nm.

### Determination of uridine in blood serum

Animals were randomly divided into 2 groups: 1—control (normoxia) rats were treated with uridine (control, *n* = 6); 2—rats with experimental AMI treated with uridine 5 min before the LCA occlusion (*n* = 6). Uridine was given i.v. at a dose of 30 mg/kg. Blood was collected from the femoral vein, and the concentration of uridine in rat blood serum was measured at the following time points: before uridine (0 min, background level), 5, 10, 15, 30, and 65 min after its administration. Serum uridine levels were determined by HPLC on an Orlant microcolumn liquid chromatograph (Medicant LLC, Russia) with a Nukleosil 5C 18 column (Macherey–Nagel GmbH & Co, Germany). Detection was fulfilled at 254 nm.

### Analysis of heart rhythm

The analysis of the incidence and severity of ischemic and reperfusion ventricular tachyarrhythmias was performed in accordance with Lambeth Conventions^63^, which are guidelines for the recognition and evaluation of experimentally induced disturbances of the cardiac rhythm. The following parameters were measured: 1, the frequency of occurrence of ventricular tachyarrhythmia; 2, the incidence of ventricular tachycardia (VT), the total duration (s), and the number of its episodes; 3, the frequency of occurrence and the total duration of episodes of ventricular fibrillation (VF), including the number of cases of the persistent form of VF, which lead to the animal death; 4, integrated assessment of the severity of reperfusion-induced rhythm disturbances in scores according to^29^: 0 score = no arrhythmia; 1 score =  ≤ 10 s VT, no VF; 2 scores = 11–30 s VT, no VF; 3 scores = 31–90 s VT, no VF; 4 scores = 91–180 s VT and/or ≤ 10 s reversible VF; 5 scores =  ≥ 180 s VT and/or ≥ 10 s reversible VF; 6 scores = lethal VF.

### Statistical analysis

The data are expressed as the mean ± standard derivation (m ± SD). Statistical analysis of the data was carried out using the GraphPad Prism version 6.0 software (GraphPad Software, CA, USA). The data were analyzed for normal distribution (Shapiro–Wilk test). The statistical significance of differences between the experimental groups was evaluated using the Fisher's Exact test or one-way repeated analysis of variance (ANOVA) followed by the Turkey multiple comparison Post Hoc test (for multiple-group comparisons). Significant differences were represented as follows: ns, not significant; *p < 0.05; **p < 0.01; ***p < 0.001 (vs. AMI or I/R group); ^#^*p* < 0.05; ^##^*p* < 0.01; ^###^*p* < 0.001 (vs. AMI (or I/R) + uridine group).

## Supplementary Information

Supplementary Information.

## Data Availability

The datasets generated during and/or analysed during the current study are available from the corresponding authors on reasonable request.
