# Supplementary material for: Uridine treatment prevents myocardial injury in rat models of acute ischemia and ischemia/reperfusion by activating the mitochondrial ATP-dependent potassium channel
Source: Sci Rep. 2021 Aug 20;11:16999. doi: 10.1038/s41598-021-96562-7 (PMC8379228; doi:10.1038/s41598-021-96562-7)
Supplement: Supplementary file 1 — Supplementary Information. [file 41598_2021_96562_MOESM1_ESM.pdf]

Supplemental Materials

to the manuscript of "Uridine treatment prevents myocardial injury in rat models of acute ischemia and ischemia/reperfusion by activating the mitochondrial ATP-dependent potassium channel" [Submission ID 41073424-7504-4e7c-97b0-6ca279847312] by I.B. Krylova, E.N. Selina, V.V. Bulyon, O.M. Rodionova, N.R. Evdokimova, N.V. Belosludtseva, M.I. Shigaeva, and G.D. Mironova

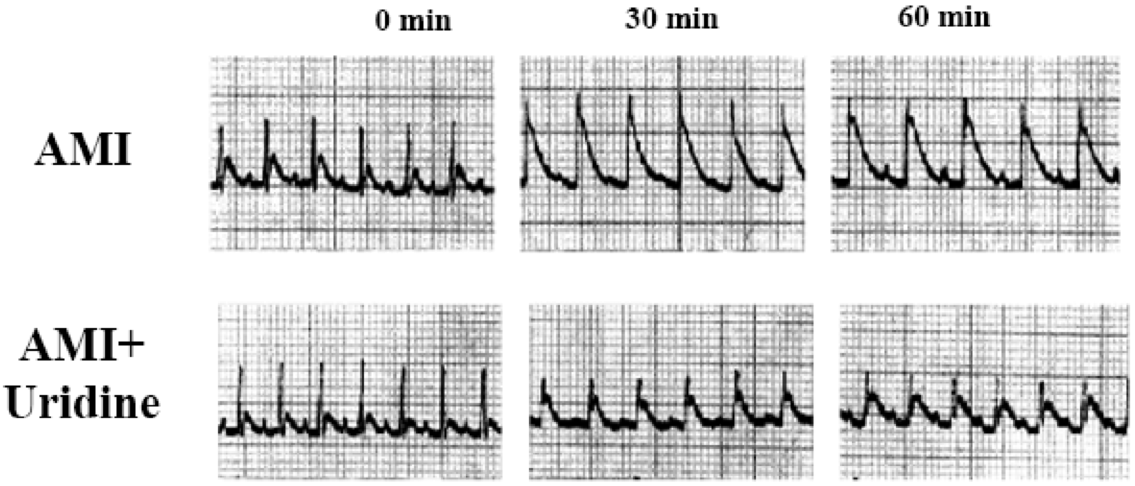

Supplementary Figure S1. Representative ECG recordings obtained at time points of 0 min (baseline), 30 min, and 60 min after the left coronary artery occlusion in the AMI rat model. Effect of uridine on the T-wave amplitude is shown. Uridine was injected 5 min before occlusion at a dose of 30 mg/kg, intraperitoneally.

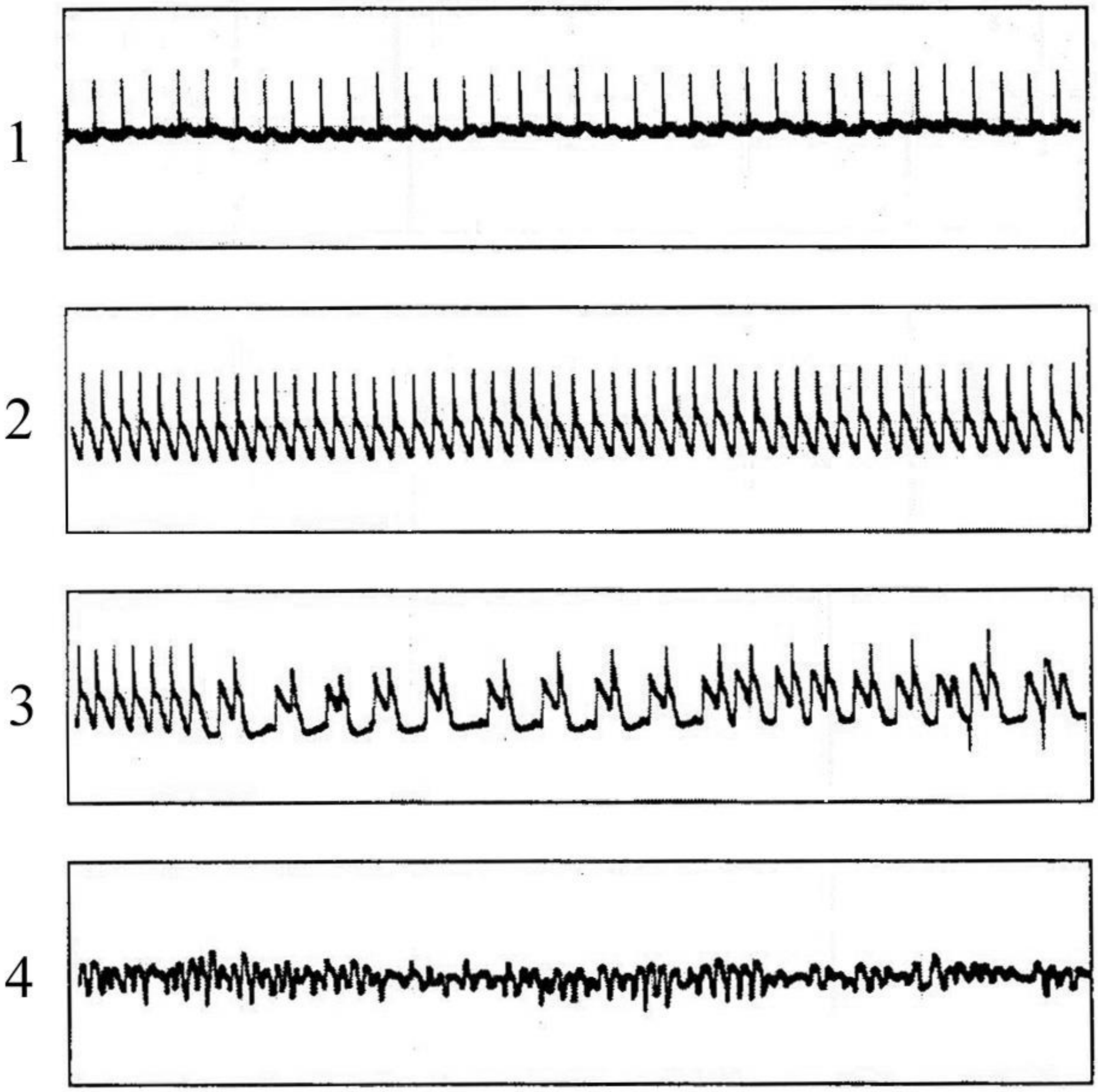

Supplementary Figure S2. Heart rhythm disorders after the LCA occlusion in the AMI rat model (ECG, lead II): 1 – sinus rhythm before occlusion; 2 – premature ventricular beats; 3 – ventricular tachycardia; 4 – ventricular fibrillation.

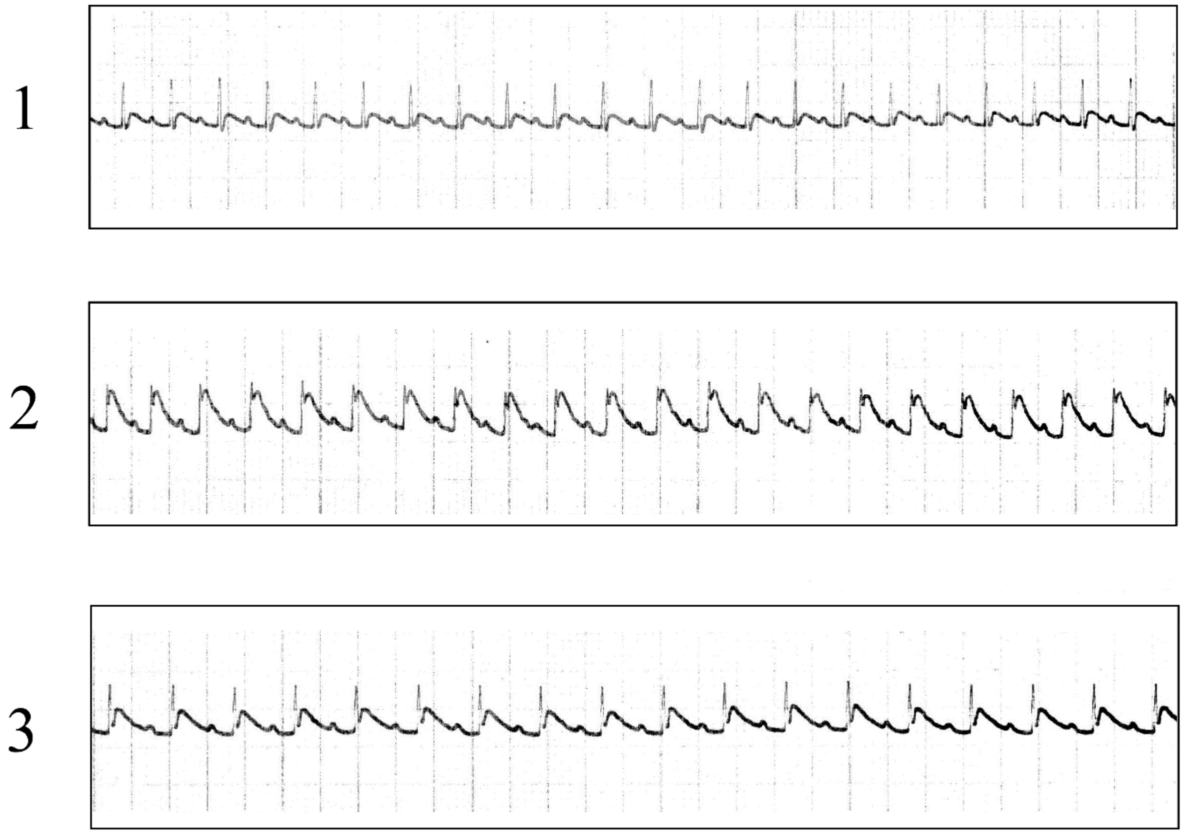

Supplementary Figure S3. Representative ECG recordings at the different time points in the I/R rat model: 1 – baseline (before I/R); 2 – 30 min of ischemia; 3 – 120 min of reperfusion.

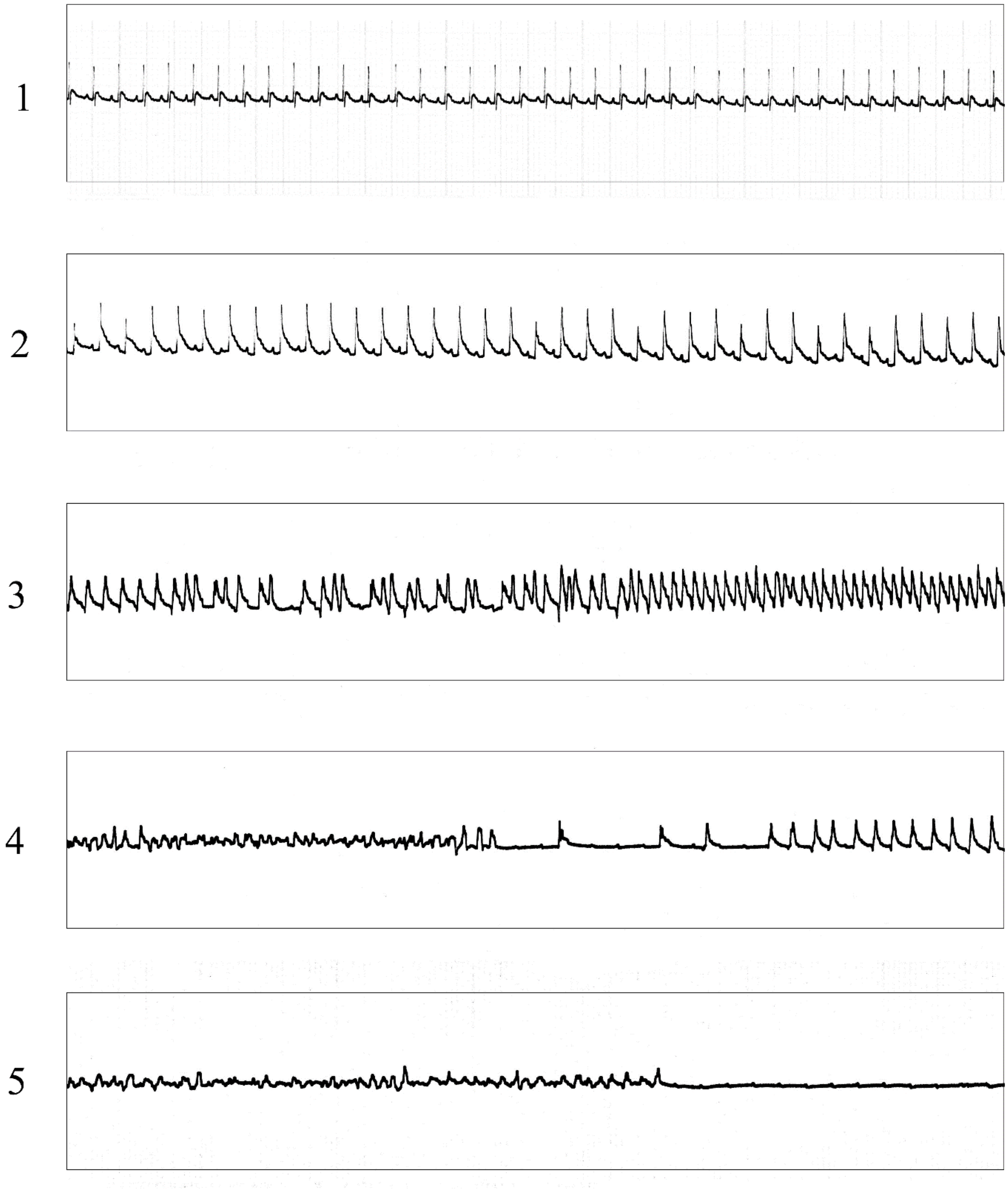

Supplementary Figure S4. Heart rhythm disorders during reperfusion arrhythmias in the I/R rat model: 1 – baseline (before I/R); 2 – 7 min of ischemia; 3 – premature ventricular beats and ventricular tachycardia; 4 – ventricular fibrillation (VF) followed by restoration of the sinus rhythm; 5 – VF followed by asystole.
